# EST Express: PHP/MySQL based automated annotation of ESTs from expression libraries

**DOI:** 10.1186/1471-2105-9-186

**Published:** 2008-04-10

**Authors:** Robin P Smith, William J Buchser, Marcus B Lemmon, Jose R Pardinas, John L Bixby, Vance P Lemmon

**Affiliations:** 1 The Miami Project to Cure Paralysis, University of Miami Miller School of Medicine, Miami, USA; 2 Neuroscience Program, University of Miami Miller School of Medicine, Miami, USA; 3 Department of Pharmacology, University of Miami Miller School of Medicine, Miami, USA; 4 Department of Neurological Surgery, University of Miami Miller School of Medicine, Miami, USA; 5 Egea Biosciences, La Jolla, USA

## Abstract

**Background:**

Several biological techniques result in the acquisition of functional sets of cDNAs that must be sequenced and analyzed. The emergence of redundant databases such as UniGene and centralized annotation engines such as Entrez Gene has allowed the development of software that can analyze a great number of sequences in a matter of seconds.

**Results:**

We have developed "EST Express", a suite of analytical tools that identify and annotate ESTs originating from specific mRNA populations. The software consists of a user-friendly GUI powered by PHP and MySQL that allows for online collaboration between researchers and continuity with UniGene, Entrez Gene and RefSeq. Two key features of the software include a novel, simplified Entrez Gene parser and tools to manage cDNA library sequencing projects. We have tested the software on a large data set (2,016 samples) produced by subtractive hybridization.

**Conclusion:**

EST Express is an open-source, cross-platform web server application that imports sequences from cDNA libraries, such as those generated through subtractive hybridization or yeast two-hybrid screens. It then provides several layers of annotation based on Entrez Gene and RefSeq to allow the user to highlight useful genes and manage cDNA library projects.

## Background

The growing trend towards high-throughput science has generated a wealth of sequence information. In many instances specific subsets of mRNAs are isolated with the goal of determining differences in expression between different populations of cells. Although microarrays have been used extensively to gauge relative expression levels, many applications such as subtractive hybridization and yeast two-hybrid libraries require that an mRNA transcript simply be present for inferences to be made. To assist in the analysis of expressed sequence tags [1] and data from other types of sequencing projects, we have designed EST Express, a web-based software suite that accepts EST sequences and gene lists and performs analyses to ascertain the identity and function of genes expressed in a sample population.

## Implementation

### Software Design

EST Express uses PHP to generate dynamic HTML and Javascript. A MySQL database records sequence and analysis information in 13 relational tables. UniGene, Entrez Gene and RefSeq updates are downloaded from the NCBI FTP server through a PHP script and saved in a local folder or parsed. Several dependency modules are required for installation, including Crossmatch [2,3], NCBI's BLAST distribution [4], and the JPGraph PHP graphics library [5]. Although EST Express is designed to be run as a web server application, it can be used in standalone mode (i.e. with no connection to the internet) if a web server application is available. Setup requires the installation of two modules (BLAST and Cross_match) and the configuration of a centralized PHP settings file, but is relatively straightforward.

### Data Analysis and Reports

#### Data Pipeline

**Figure 1:**
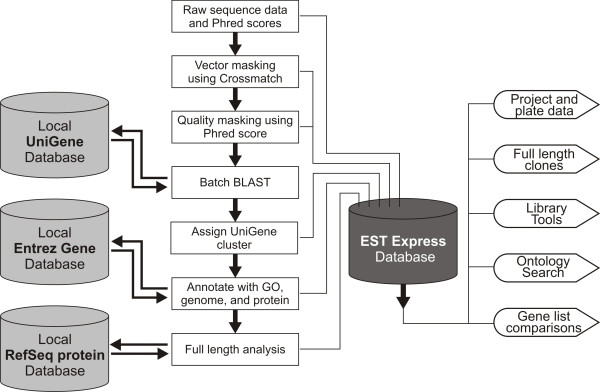
**Data Pipeline**. Raw sequence data is imported into EST Express along with phred scores, where it is then screened for contaminating vector sequences and masked for quality. Good quality sequences are then batch BLASTed against a local UniGene database and the top hit is assigned to each sample. A local copy of the Entrez Gene database is then linked to the UniGene identifier and used to annotate each sequence with a description, Gene Ontology identifiers, RefSeq mRNA and protein links, and genomic context. Oligo(dT)-primed sequences can then be analyzed for full-length status using a local copy of the RefSeq protein database and the Entrez Gene cross references. The user interface then provides several ways to browse and visualize the results from the pipeline.

EST Express accepts base calls and Phred scores in FASTA format, which it then parses and screens for user provided contaminating vector sequence using Crossmatch [2,3] (See Figure 1). Phred scores are then used to define a window within the sequence that is suitable for BLASTing. Sequences without high (>20) Phred scores are designated low sequence reads, and those with predominantly vector sequence are designated vector-only. The remaining sequences are then subjected to a similarity search against a local copy of the UniGene database using BLASTN. The top cluster from each BLAST result is stored and linked to the sample sequence. The "gene2unigene" conversion table produced by NCBI [6] is then used to link UniGene clusters with the Entrez Gene database for further annotation. To simplify the annotations of those identifiers that have many-to-many relationships, EST Express builds a second table named "unigeneprefs" which selects the best Entrez Gene ID for each UniGene entry based on the relative degree of annotations (e.g. descriptive naming, mRNA link, protein link). Other analyses listed below are then performed on the combined data and linked back to the sample.

#### Data Representation

**Figure 2:**
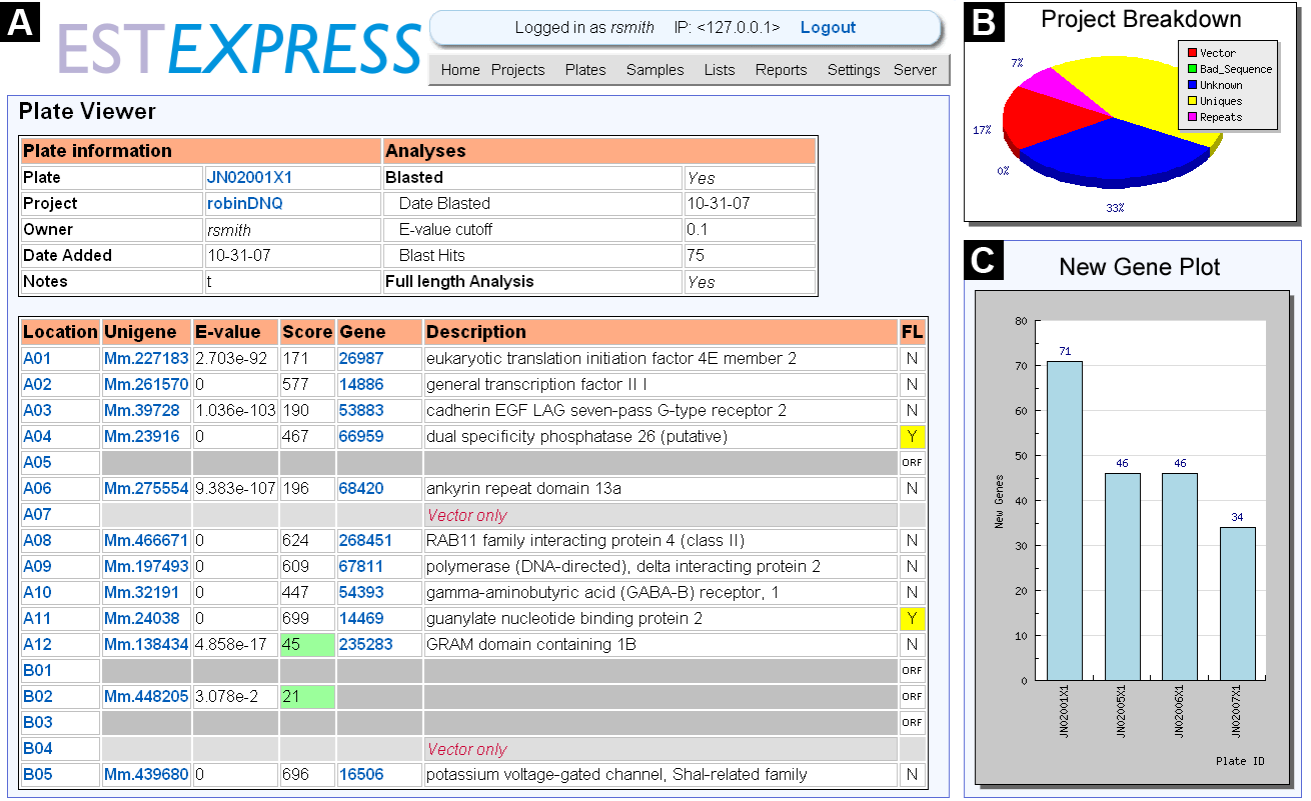
**Screenshots from EST Express**. *A: *Screenshot of the "Plate Viewer" page showing details for plate JN02001X1 owned by user "rsmith" in project "robinDNQ". For each matched sample in the plate a UniGene identifier is listed, along with the BLAST score and Entrez Gene and full-length annotations. *B: *Capture from the "Project Viewer" page showing a graphical breakdown of ESTs within a project. "Vector" refers to sequences designated vector-only. "Bad_sequence" refers to sequences with low quality sequence reads. "Unknown" refers to samples that are neither vector-only nor low quality, but do not match against the UniGene database. "Uniques" refers to the number of unique UniGene clusters in the project and "Repeats" refers to additional instances of those unique clusters. *C: *Capture from the "New Gene" library tool, showing the number of new unique UniGene clusters found with each successive round of sequencing. Further rounds of sequencing produce progressively fewer unique clusters. Both *B *and *C *were produced dynamically using the JPGraph PHP graphics library.

Sequences imported into EST Express are represented as "samples" (Figure 2a) and linked to different analyses through unique identifiers. Each sample is, in turn, part of a "plate", which encompasses all samples that were part of the original imported sequence file. Each plate then belongs to an overall "project" (Figure 2b), which possesses functional characteristics that make it distinct. This structure was adopted because of the nature of sequencing projects – often 96 or 384 well plates are sequenced in succession as part of a larger project. Analyses such as batch BLAST can be performed on individual plates or on an entire project.

#### Sample Identification

Once samples have been loaded into a project, the underlying goal is to assign them a UniGene cluster and a resulting Entrez Gene ID, which provides access to the vast collection of annotations available through the Entrez Gene database. Because this requires that a UniGene cluster database be available, the EST Express frame-work is most relevant for projects involving model organisms (of which there were 74 at the time of writing). Sequences from non model organisms can also be identified provided they have sufficient sequence similarity with those of a model organism.

#### Entrez Gene Annotations

The Entrez Gene database [7] is a central depot for gene-specific information. EST Express makes full use of the annotations contained within, linking UniGene cluster IDs to Entrez Gene IDs. Because of the large size of the Entrez Gene database (>600MB for the Mus_musculus version alone) there is considerable interest in developing utilities that can parse the provided ASN.1 files into a useable format [8]. Many of the Entrez Gene annotations, however, can also be found in flat text files [6], which are much easier to parse. Four of these files (gene_info, gene2unigene, gene2go and gene2refseq) are downloaded by EST Express and combined into a single MySQL table within minutes. Users can then search annotations that match to samples using the search tool.

#### Full-length Analysis

In many cases it is desirable to know whether a library clone contains the full open reading frame for the gene in question. This allows for selected full-length clones to be re-arrayed and used in a variety of expression studies. EST Express can carry out such an analysis for Oligo(dT)-primed cDNAs that have sequence reads from the 5' end. Once a sample sequence has been identified, the corresponding RefSeq protein ID is extracted from the Entrez Gene table and matched against a locally downloaded copy of the RefSeq protein database. The EST is then translated into three different frames and matched against the first 10 amino acids of the protein sequence. Using this comparison, each annotated sequence is assigned "full-length" or "not full-length" status. Samples that are not annotated with a RefSeq protein identifier are examined for long open reading frames, the results of which are stored and can be queried for further analysis.

#### Library Tools

EST Express offers two tools that enable the user to assess the content of the source library being sequenced. The first tool generates a graph of the number of novel UniGene clusters found in each successive sequenced plate added to a project (Figure 2c). This feature is a useful indicator of library complexity as well as of how many sequences the user can expect to obtain. The second tool reports the number of times each UniGene cluster has been found within a project. This is a useful measure for subtracted libraries because cDNAs sampled more frequently correspond to transcripts that are enriched in the tester mRNA pool.

#### Gene Lists

Thus far, no individual technique provides complete information about the genes that are at work in a system. It is therefore often useful to compare lists of genes for commonalities or differences. EST Express allows the user to generate a list of sample IDs, UniGene clusters or Entrez Gene IDs from a project or plate based on specific criteria. Lists of identifiers may also be uploaded as a text file originating from another experiment (e.g. microarray, mass spectrometry). Once a list is created it can be compared against one or more lists, the results of which can be saved as a new list. Each list can then be exported with full Entrez Gene annotations to an Excel spreadsheet for further analysis.

## Results and Discussion

### Evaluation with subtracted library sequences

**Figure 3:**
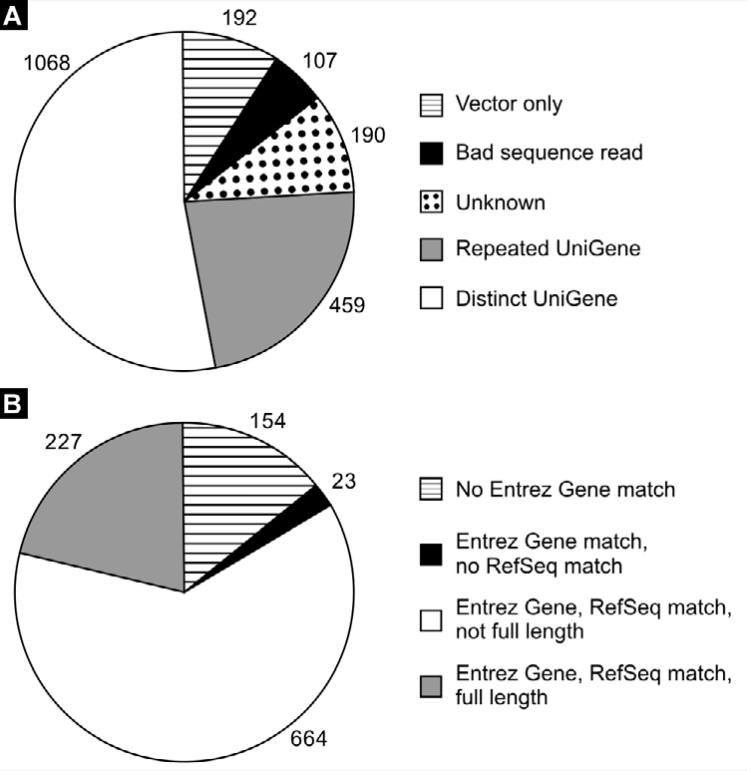
**Results of analyses on the subtracted data set**. *A: *Distribution of identifications made by EST Express for all 2,016 samples. *B*: Distribution of associations made for 1,068 distinct UniGene entries.

EST Express has been successfully implemented and used to identify and annotate 4 separate libraries containing over 2,500 samples. Of these four libraries, the largest is the "subtracted" library generated through subtractive hybridization of tissue specific genes. For this library, 21 plates containing 2,016 samples were analyzed, resulting in 1,068 unique cDNAs (See Figure 3a). Of the 2,016 samples, 192 were vector-only sequences and 107 were low quality sequence reads. Of the 1,068 unique cDNAs, 914 matched Entrez Gene entries. Selection of appropriate Entrez Gene identifiers based on RefSeq links proved efficacious: only 23 sequences match Entrez Gene identifiers without a RefSeq link, allowing full-length analysis of 83% of samples returning a BLAST hit (Figure 3b). Of those samples that were analyzed, 227 were found to be full-length.

### Comparison to related software packages

EST Express is similar in broad terms to other sequence pipeline software packages, including PipeOnline 2.0 [9], ESTAP [10], EST-PAGE [11] and ESTIMA [12]. However, there are several key differences that make EST Express an attractive alternative to the bioinformatics community.

EST Express is written entirely in PHP, an open source scripting language that is platform independent and extremely popular amongst web developers. All four of the packages listed above are Perl based and could not be installed on Windows based server without modifications. EST Express uses the MySQL database platform for storage of sequence data and analyses. MySQL is also open source and freely available under the GPL, contrasting with the commercial package Oracle, which is employed by ESTAP [10] and ESTIMA [11]. Unlike PipeOnline 2.0 [9], EST Express is also freely available for download and installation, and is distributed with explicit instructions for both Linux and Windows based machines.

The central difference between EST Express and these other packages is that it was designed for a post genome world in which researchers have the ability to generate specialized expression libraries and require a pipeline for identifying the mRNAs within. EST Express is unique in that it has a built-in support for identifying full-length cDNAs, diagnostic tools for gauging the complexity of the cDNA library, gene list tools for comparisons with microarray data and convergence of annotations through the use of the relatively recent Entrez Gene database [7].

### Potential applications

Although EST Express was primarily developed to analyze libraries generated by subtractive hybridization, it could be employed in any number of applications, some of which are outlined below:

a) Generic libraries in which the host organism has an established UniGene cluster database.

b) Libraries generated through subtractive hybridization of two or more mRNA populations

c) Screened yeast two-hybrid prey libraries

d) Comparison of gene lists generated on different platforms

e) Annotation of custom gene lists with terms from the Entrez Gene database

## Conclusion

We have developed a valuable new tool named EST Express for the identification, annotation and analysis of cDNA library sequences. EST Express is unique in that it is cross-platform, is freely available, makes full use of annotations from the Entrez Gene database and allows the user to assess the state of the cDNA library using diagnostic tools. EST Express is available under the GNU General Public License [13] and may be downloaded from its project website [14].

## Availability and Requirements

• **Project name: **EST Express

• **Project home page: **

• **Operating system(s): **Windows NT/2000/XP, Linux, potentially others

• **Programming language: **PHP/MySQL

• **Other requirements: **NCBI BLAST Toolkit, Crossmatch, JPGraph library

• **License: **GNU General Public License [13]

• **Any restrictions to use by non-academics: **Licence required

## List of Abbreviations

BLAST: Basic Local Alignment Search Tool; cDNA: Complementary Deoxyribonucleic Acid; EST: Expressed Sequence Tag; FTP: File Transfer Protocol. GPL: GNU General Public License; HTML: Hypertext Markup Language. ID: Identifying number; GUI: Graphical User Interface; mRNA: Messenger Ribonucleic Acid; MySQL: My Structured Query Language; PHP: PHP Hypertext Processor.

## Authors' contributions

RS wrote the code for the software package, developed the project website and documentation, and prepared the manuscript. WB and ML participated in the testing and development of the software and contributed to the manuscript and software manual. JP, JB and VL provided insights on software development and testing and critically reviewed the manuscript.
